# Ezrin, radixin, and moesin are novel citrullinated proteins in the decidua during pregnancy^†^

**DOI:** 10.1093/biolre/ioaf241

**Published:** 2025-10-27

**Authors:** Kouhei Yamashita, Shinji Ito, Junko Satoh, Akifumi Takaori-Kondo, Kiyomi Mizugishi

**Affiliations:** Department of Hematology, Kyoto University Hospital, Sakyo-ku, Kyoto 606-8507, Japan; Medical Research Support Center, Graduate School of Medicine, Kyoto University, Sakyo-ku, Kyoto 606-8501, Japan; Medical Research Support Center, Graduate School of Medicine, Kyoto University, Sakyo-ku, Kyoto 606-8501, Japan; Department of Hematology, Kyoto University Hospital, Sakyo-ku, Kyoto 606-8507, Japan; Department of Hematology, Kyoto University Hospital, Sakyo-ku, Kyoto 606-8507, Japan

**Keywords:** citrullination, decidualization, ezrin/radixin/moesin (ERM), peptidylarginine deiminase, pregnancy, sphingosine kinase

## Abstract

For a successful pregnancy, decidualization is a crucial process involving the significant transformation of endometrial stromal cells surrounding the implanting blastocysts. Disruption of this process can lead to the breakdown of the fetomaternal interface and result in early pregnancy loss. However, the precise mechanisms governing this process remain incompletely understood. This study aimed to elucidate the impact of citrullination, a post-translational modification, on decidualization in mice and humans. Immunohistochemical analysis and immunoprecipitation followed by immunoblotting were performed on mouse and human uterine tissues. In this study, we showed that ezrin/radixin/moesin (ERM) proteins are expressed in decidual and endothelial cells within the decidua during pregnancy, with their expression patterns significantly influenced by two factors: citrullination mediated by peptidylarginine deiminase enzymes and sphingosine kinase activity. Notably, ERM proteins were found to undergo citrullination exclusively in the decidua during pregnancy, but not in interimplantation tissues during pregnancy or in nonpregnant uteri in mice. Similar findings were observed in human decidual tissues from cases of spontaneous abortion and elective termination. In conclusion, this study demonstrates that ERM proteins undergo citrullination in the decidua during early pregnancy in both mice and humans. Our findings provide important insights into the molecular changes involved in pregnancy, particularly decidualization.

## Teaser

Ezrin/radixin/moesin (ERM) proteins undergo citrullination in the decidua during early pregnancy in mice and humans.

## Graphical Abstract

**Graphical Abstract:**
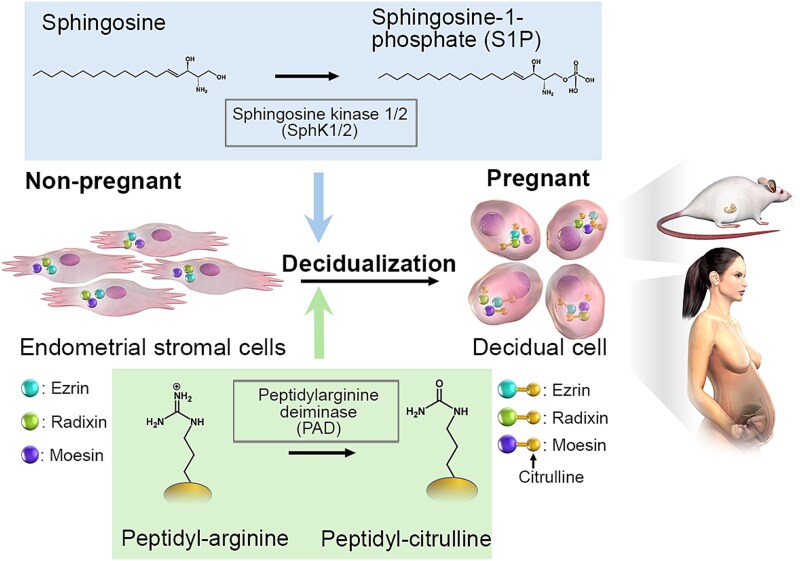

## Introduction

The sphingolipid metabolic pathway produces bioactive signaling molecules, including sphingosine-1-phosphate (S1P), a versatile extracellular phospholipid messenger. Sphingosine-1-phosphate exerts its effects by activating a group of cell-surface G protein-coupled receptors, designated S1PR1-5 [1]. Recent research has identified S1P as a critical regulator in various physiological and pathological processes, such as immunity [2], cancer [3], and inflammation, particularly in ulcerative colitis [4, 5] and autoimmune diseases [6], through its interaction with S1P receptors. Sphingosine-1-phosphate is synthesized by two sphingosine kinases, *Sphk1* and *Sphk2*, through adenosine triphosphate-dependent phosphorylation of sphingosine [7, 8].

Genetic studies have shown that simultaneous deletion of *Sphk1* and *Sphk2* (*Sphk1*^−/−^*Sphk2*^−/−^) on a mixed genetic background (C57BL/6 × 129Sv) results in embryonic lethality around day 11.5 postcoitum (pc) due to significant defects in neurogenesis and angiogenesis. In contrast, mice lacking only *Sphk1* or *Sphk2* exhibit no obvious morphological or functional abnormalities [9, 10].

We further investigated the role of *Sphk* in female reproduction. Lipid mediators, including prostaglandins, lysophosphatidic acid, and cannabinoids, are known to play critical roles in early pregnancy processes such as embryo implantation and decidualization. Disruption of these pathways has been linked to various abnormal pregnancy outcomes in rodent models and potentially in humans [11]. Our previous study showed that female *Sphk1*^−/−^*Sphk2*^+/−^ mice on a 129Sv/C57BL/6 mixed background exhibited reproductive abnormalities [12]. During normal pregnancy, the sphingolipid metabolic pathway was found to be highly active in the decidua. However, in *Sphk1^−/−^Sphk2^+/−^* uteri, defective decidualization led to decidual cell death and extensive rupture of decidual blood vessels, resulting in maternally driven early pregnancy loss [12]. Additionally, another study reported that three subunits of serine palmitoyltransferase (SPT), the initial key enzymes in sphingolipid biosynthesis, are significantly upregulated in mouse uterine stromal cells during decidualization. Inhibition of SPT was shown to impair the decidualization process [13]. Together, these findings highlight the essential role of the sphingolipid metabolic pathway in decidualization, with pathway disruption leading to defective decidualization and early pregnancy loss in mice.

*Sphk1*  ^−/−^*Sphk2*  ^+/−^ female mice exhibited abnormally elevated expression of the neutrophil chemoattractants CXCL1 and CXCL2 in the decidual region surrounding the embryo. This increase facilitated extensive neutrophil infiltration into the fetomaternal interface, leading to maternally driven early pregnancy loss [14]. Inhibiting neutrophil infiltration alleviated pregnancy loss in *Sphk1*^−/−^*Sphk2*^+/−^ female mice. Similarly, in primary human decidual cells, *Sphk* deficiency significantly enhanced the secretion of neutrophil chemoattractants, including CXCL1 and IL-8, by decidual cells [14]. These findings suggest that neutrophil-mediated tissue damage plays a crucial role in *Sphk*-related pregnancy loss.

We also identified excessive neutrophil extracellular trap (NET) formation as a key factor contributing to pregnancy loss in *Sphk*-deficient mice [15]. Neutrophil extracellular traps, first described in 2004, are extracellular structures composed mainly of DNA fibers, histones, and antimicrobial granule proteins such as neutrophil elastase and myeloperoxidase, which are used to trap microbes [16]. Histone hypercitrullination, a process in which histone arginine residues are converted to citrulline residues by peptidylarginine deiminase 4 (PADI4) in a calcium-dependent manner, is essential for chromatin decondensation and subsequent NET formation [17–19]. Although originally identified as a novel immune defense mechanism, NETs have also been implicated in various noninfectious conditions. Excessive NET formation has been associated with the development of systemic autoimmune and autoinflammatory diseases [20] as well as cardiovascular diseases [21]. Aberrant NET formation has also been reported in pregnancy-related disorders [22], including preeclampsia [23], antiphospholipid syndrome [24, 25], and preterm birth [26]. Our previous study showed that pregnant *Sphk1^−/−^Sphk2^+/−^* female mice exhibit excessive NET formation at the fetomaternal interface, characterized by histone hypercitrullination and overexpression of PADI4 in neutrophils [15]. Inhibiting NET formation with a PADI4 inhibitor partially restored normal embryonic development in these mice. These findings suggest that NETs play a crucial role in decidual tissue damage in *Sphk1^−/−^Sphk2^+/−^* female mice, contributing to early pregnancy loss.

Ezrin, radixin, and moesin (ERM) proteins are adaptor molecules that connect the actin cytoskeleton to the plasma membrane and play a role in regulating various physiological processes, including cell polarity, cell–cell adhesion, cell division, cell migration, endocytosis, and exocytosis [27, 28]. Ezrin, radixin, and moesin proteins exist in two conformational states. In their inactive form, ERM proteins are in a closed configuration in the cytosol. When phosphorylated at the C-terminal threonine residue, they undergo activation, linking to the cell membrane at the N-terminal domain (FERM) and the cytoskeleton at the C-terminal domain (C-ERMD). Notably, sphingolipids are closely involved in regulating ezrin. Sphingosine-1-phosphate promotes ezrin phosphorylation, leading to activation, while ceramide is essential for ezrin deactivation [29].

We hypothesized that PADI-mediated citrullination may influence the function of proteins involved in decidualization. Given the emerging connection between sphingolipids, ERM proteins, and PADI enzymes/citrullination led us to explore the role of PADI/citrullination in normal pregnancy and *Sphk*-mediated pregnancy loss using *in vivo* mouse models and human tissues.

## Materials and methods

### Study approval

All animal studies were conducted in accordance with protocols approved by Kyoto University (Approval number: Med kyo 24144). Studies involving human tissues were approved by the Institutional Review Board at Kyoto University (Approval number: R0575-2). Written informed consent was obtained from both the patients and their spouses.

### Animals

The *Sphk1* and *Sphk2* genes were knocked out as previously described [9, 10]. Mice were maintained on a mixed genetic background (C57BL/6 × 129Sv). For immunoblot analysis, mice on a C57BL/6 background were also used. In all experiments, 6–12-week-old female mice were mated with wild-type males. The day a vaginal plug was observed was designated as day 0.5 pc.

### Sample preparation

In mouse experiments, nonpregnant whole uteri, deciduas, and interimplantation tissues from day 6.5 pc, day 7.5 pc, day 8.5 pc, or day 9.5 pc uteri (from which embryos had been removed) were immediately frozen. In human experiments, decidual specimens from spontaneous abortions or elective terminations of first-trimester pregnancies (7–9 weeks) in healthy individuals aged 33–34 years were analyzed (*n* = 3). The specimens were collected via vaginal curettage. Clinical data on the patients are as follows: Case 1, a 34-year-old patient with spontaneous abortion of an 8-week first-trimester pregnancy; Case 2, a 34-year-old patient with elective termination of a 9-week first-trimester pregnancy; Case 3, a 33-year-old patient with elective termination of a 7-week first-trimester pregnancy.

### Histological analysis

Uteri were fixed in 10% formalin and embedded in paraffin. Serial sections (5 μm) were prepared at 10–30 μm intervals and stained with hematoxylin and eosin for general morphological analysis. Paraffin sections were deparaffinized and rehydrated. Antigen retrieval was performed by incubating the samples with Target Retrieval Solution (Agilent Technologies, Santa Clara, CA, USA) at 95°C for 20 min. For immunoperoxidase staining, endogenous peroxidase activity was blocked by incubating the specimens with 1% hydrogen peroxide in water for 30 min. The specimens were then incubated overnight at 4°C with rabbit anti-PADI2 (Proteintech, Rosemont, IL, USA, #12110-1-AP, RRID: AB_2159475), rabbit anti-PADI4 (Proteintech, #17373-1-AP, RRID: AB_2878398), rabbit anti-radixin (Abcam, Cambridge, UK, #ab52495, RRID: AB_882259), rabbit anti-ezrin (Abcam, #ab41672, RRID: AB_941504), rabbit anti-moesin (Abcam, #ab52490, RRID: AB_881245), or rabbit anti-ezrin (pThr567)/radixin (pThr564)/moesin (pThr558) (Abcam, #ab76247, RRID: AB_1523584) antibodies at a 1:100 dilution. The specimens were then incubated with biotin-conjugated goat anti-rabbit immunoglobulin G (IgG) for 30 min at room temperature. Furthermore, the specimens were incubated overnight at 4°C with biotinylated anti-*Dolichos biflorus* agglutinin (DBA) (Vector Laboratories, Newark, CA, USA, #B1035, RRID: AB_2314288) antibody at a 1:500 dilution to label decidual natural killer (dNK) cells. The avidin/biotin horseradish peroxidase system (Vector Laboratories) with diaminobenzidine was used for visualization. Images were captured using an Eclipse E600 microscope (Nikon, Tokyo, Japan) with a DP21 camera (Olympus, Tokyo, Japan). The magnification for each image is specified in the figure legend for each experiment.

###  Peptidylarginine deiminase inhibitor treatment

Female mice were administered intraperitoneal injections of the PADI inhibitor Cl-amidine (100 mg/kg/day) (Merck Millipore, Burlington, MA, USA) dissolved in dimethyl sulfoxide (DMSO) and further diluted with normal saline, on days 5 and 7 of pregnancy. Control mice were treated with DMSO vehicle diluted with normal saline. The optimal dose and frequency of Cl-amidine that provided the best therapeutic effects with minimal side effects were determined. Mice were sacrificed on day 7.5 of pregnancy, and their uteri were dissected for histological analysis.

### Immunoprecipitation

Mouse uteri were homogenized in T-PER tissue protein extraction reagent (Thermo Fisher Scientific, Waltham, MA, USA) with a complete protease inhibitor cocktail (Sigma Aldrich, Saint Louis, MO, USA). Homogenates from day 7.5 pc deciduas, day 7.5 pc interimplantation tissues (uterine tissues without implantation sites), and nonpregnant uterine tissues from wild-type and *Sphk1^−/−^Sphk2^+/−^* female mice on mixed (C57BL/6 × 129Sv) or C57BL/6 backgrounds were used for immunoprecipitation experiments (*n* = 3–5 per group). Additionally, 50 μl of Dynabeads (Thermo Fisher Scientific, #DB10003) and 5 μl of mouse anti-citrulline antibody (Cayman Chemical, Ann Arbor, MI, USA, #30773) were added to 2.5 mg of each homogenate, and the mixture was incubated for 20 min in a rotating chamber. The beads were washed three times with phosphate-buffered saline (PBS). The coprecipitated proteins were eluted in sodium dodecyl sulfate (SDS) sample buffer and analyzed by immunoblotting. Human decidual tissues were analyzed using the same method as mouse uteri.

### Immunoblotting

Proteins were separated by SDS-polyacrylamide gel electrophoresis and transferred to nitrocellulose membranes using the XCell SureLock Mini-Cell (Thermo Fisher Scientific). The membranes were blocked with Blocking One (Nacalai Tesque, Kyoto, Japan) overnight at 4°C and then incubated with primary antibodies in the blocking buffer for 1 h. The primary antibodies used were rabbit anti-radixin (Abcam, #ab52495), rabbit anti-ezrin (Abcam, #ab270442), rabbit anti-moesin (Abcam, #ab52490), rabbit anti-vimentin (Abcam, #ab92547, RRID: AB_10562134), rabbit anti-beta actin (Proteintech, #20536–1-AP, RRID: AB_10700003), and anti-ezrin (pThr567)/radixin (pThr564)/moesin (pThr558) (Abcam, #ab76247) at a 1:500 dilution. The blots were washed three times with PBS-T (with 0.1% Tween 20) and incubated with peroxidase-conjugated goat anti-rabbit IgG (Santa Cruz Biotechnology, Dallas, TX, USA, #sc-2004, RRID: AB_631746) in the blocking buffer for 1 h. Chemiluminescence detection was performed using Chemi-Lumi One Super (Nacalai Tesque).

### Blocking of phosphorylated ezrin, radixin, and moesin proteins

Female mice were injected intraperitoneally with 0.02 mg of anti-ezrin (pThr567)/radixin (pThr564)/moesin (pThr558) antibody (Abcam, #ab76247, RRID: AB_1523584) diluted in PBS on day 4 or on both days 4 and 5 pc. Mice were sacrificed on day 7.5 of pregnancy, and their uteri were collected for histological analysis. Additionally, uteri and embryos were examined on day 14.5 pc. Control mice received intraperitoneal injections of PBS.

## Results

### PADI2 and PADI4 are complementarily expressed in the decidua

**Figure 1:**
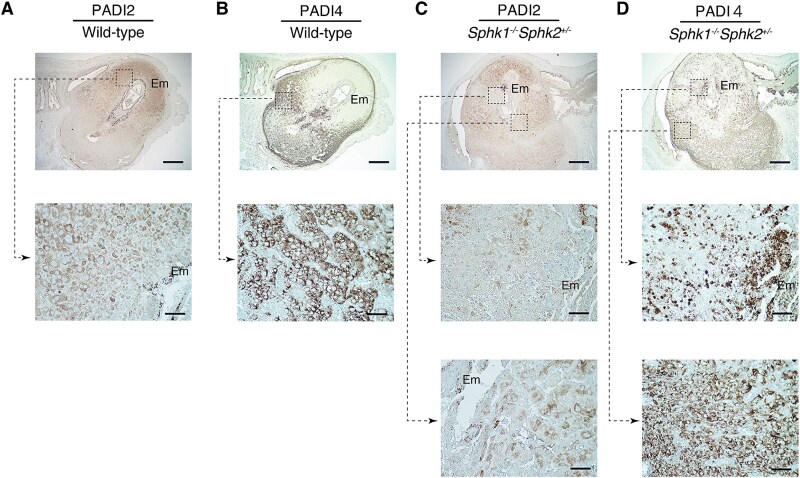
Complementary expression patterns of PADI2 and PADI4 in the decidua. (A and C) Immunostaining with anti-PADI2 antibody on day 7.5 pc wild-type (A) and *Sphk1*^−/−^*Sphk2*^+/−^ (C) uteri. (B and D) Immunostaining with anti-PADI4 antibody on day 7.5 pc wild-type (B) and *Sphk1*^−/−^*Sphk2*^+/−^ (D) uteri. The middle and lower panels show high-power views of the boxed areas from the corresponding upper panels. Em, embryo. Scale bars in panels A–D represent 500 μm (upper panels) and 50 μm (middle and lower panels). Data are representative of three independent experiments with similar results.

We initially investigated the expression patterns of PADI2 and PADI4 in the decidua of pregnant (day 7.5 pc) wild-type and *Sphk1^−/−^Sphk2^+/−^* mice with a mixed genetic background (C57BL/6 × 129Sv). Immunohistochemical analysis showed that PADI2 was specifically expressed in the decidual cells on the antimesometrial side of the decidua surrounding the embryos in wild-type mice (Figure 1A). In contrast, PADI4 was highly expressed in the decidual cells on the mesometrial side of pregnant wild-type mice, as previously reported (Figure 1B) [15]. In *Sphk1^−/−^Sphk2^+/−^* mice, PADI2 was also present in the decidual cells on the antimesometrial side (Figure 1C, lower panel), although its expression was barely detectable at the fetomaternal interface due to the death of decidual cells (Figure 1C, middle panel). PADI4 expression was also observed in the decidual cells of the mesometrial region in *Sphk1^−/−^Sphk2^+/−^* mice (Figure 1D, lower panel), in addition to infiltrating neutrophils at the fetomaternal interface, as previously shown (Figure 1D, middle panel) [15]. No PADI1 expression was detected in the decidual tissues of either wild-type or *Sphk1^−/−^Sphk2^+/−^* mice (data not shown). These findings highlight the complementary expression patterns of PADI2 and PADI4 in the decidua during pregnancy and suggest a potential role for PADI enzymes in decidualization.

### Inhibition of peptidylarginine deiminase and Sphk activity significantly changed the expression patterns of ezrin, radixin, and moesin in the decidua during pregnancy

**Figure 2:**
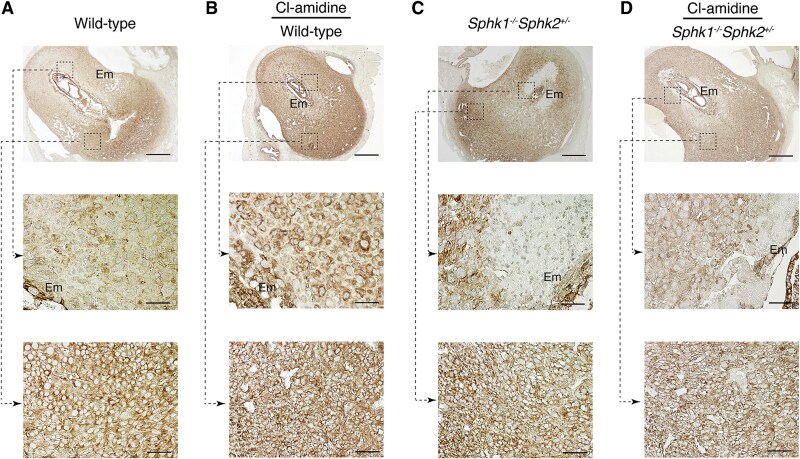
Radixin expression in the decidua during pregnancy. (A and C) Immunostaining with anti-radixin antibody on day 7.5 pc wild-type (A) and *Sphk1*^−/−^*Sphk2*^+/−^ (C) uteri. (B and D) Immunostaining with anti-radixin antibody on day 7.5 pc wild-type (B) and *Sphk1*^−/−^*Sphk2*^+/−^ (D) uteri treated with Cl-amidine. The middle and lower panels show high-power views of the boxed areas from the corresponding upper panels. Em, embryo. Scale bars in panels A–D represent 500 μm (upper panels) and 50 μm (middle and lower panels). Data are representative of three independent experiments with similar results.

To investigate the impact of PADI enzymes on the expression of ERM proteins, pregnant wild-type and *Sphk1^−/−^Sphk2^+/−^* mice with a mixed genetic background (C57BL/6 × 129Sv) were treated with Cl-amidine, a pan-PADI inhibitor, on days 5 and 7 pc, and uteri and embryos were evaluated on day 7.5 pc by immunohistochemical analysis. In untreated day 7.5 pc wild-type mice, radixin was expressed only in the decidual cells of the mesometrial region (Figure 2A, lower panel), but not in the antimesometrial region surrounding the embryo (Figure 2A, middle panel). However, Cl-amidine treatment drastically altered the expression pattern of radixin. In wild-type mice, radixin was strongly expressed in the cytoplasm of decidual cells in the antimesometrial region (Figure 2B, middle panel), in addition to the mesometrial decidual tissue (Figure 2B, lower panel) and was also observed in the mesometrial decidual cells of *Sphk1^−/−^Sphk2^+/−^* mice (Figure 2C, lower panel). However, in *Sphk1^−/−^Sphk2^+/−^* mice, radixin expression in the antimesometrial decidual cells was weaker, with no expression detected in the region around the embryo due to decidual cell death caused by extensive neutrophil infiltration at the fetomaternal interface (Figure 2C, middle panel). Treatment with the PADI inhibitor Cl-amidine reduced decidual cell death in these mice, restoring radixin expression at the fetomaternal interface (Figure 2D, middle panel).

**Figure 3:**
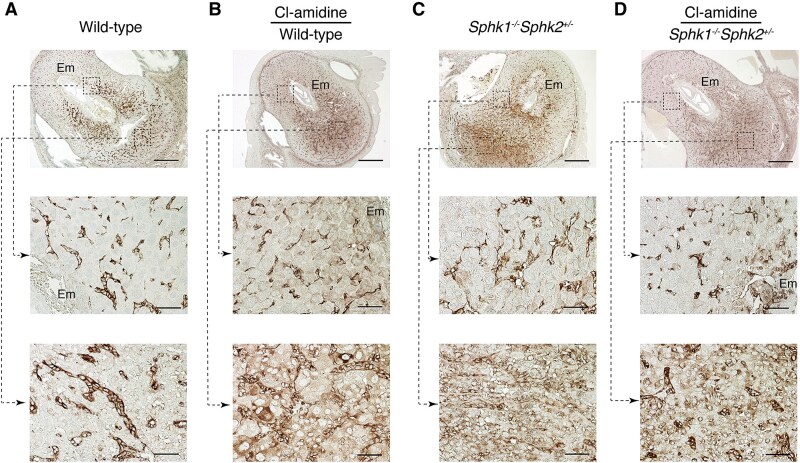
Moesin expression in the decidua during pregnancy. (A and C) Immunostaining with anti-moesin antibody on day 7.5 pc wild-type (A) and *Sphk1*^−/−^*Sphk2*^+/−^ (C) uteri. (B and D) Immunostaining with anti-moesin antibody on day 7.5 pc wild-type (B) and *Sphk1*^−/−^*Sphk2*^+/−^ (D) uteri treated with Cl-amidine. The middle and lower panels show high-power views of the boxed areas from the corresponding upper panels. Em, embryo. Scale bars in panels A–D represent 500 μm (upper panels) and 50 μm (middle and lower panels). Data are representative of three independent experiments with similar results.

We then examined the expression pattern of moesin. In day 7.5 pc wild-type mice, moesin was primarily expressed in endothelial cells on both the antimesometrial (Figure 3A, middle panel) and mesometrial sides of the decidua (Figure 3A, lower panel). Notably, PADI inhibition by Cl-amidine treatment induced the expression of moesin in decidual cells, in addition to endothelial cells, throughout the decidual region (Figure 3B, middle and lower panels). In *Sphk1^−/−^Sphk2^+/−^* mice, moesin was expressed in endothelial cells on both the antimesometrial (Figure 3C, middle panel) and mesometrial sides of the decidua (Figure 3C, lower panel). Furthermore, decidual cells across the entire decidual region expressed moesin weakly but noticeably, similar to wild-type mice treated with Cl-amidine (Figure 3C, middle and lower panels). Cl-amidine treatment had minimal impact on moesin expression in *Sphk1^−/−^Sphk2^+/−^* mice (Figure 3D).

**Figure 4:**
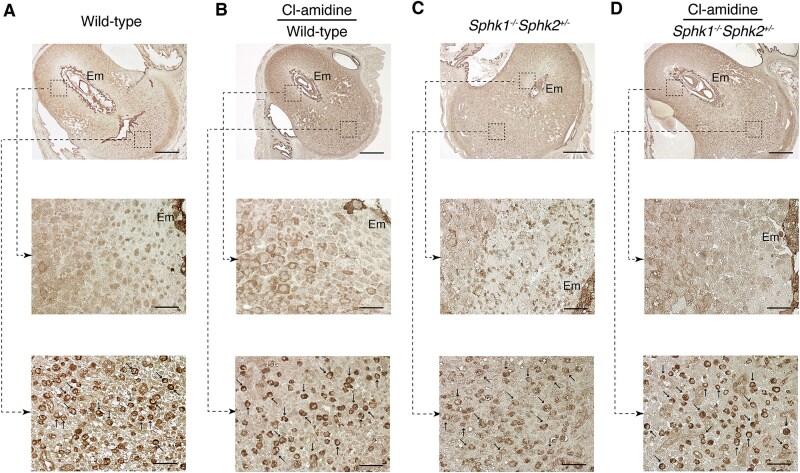
Ezrin expression in the decidua during pregnancy. (A and C) Immunostaining with anti-ezrin antibody on day 7.5 pc wild-type (A) and *Sphk1*^−/−^*Sphk2*^+/−^ (C) uteri. (B and D) Immunostaining with anti-ezrin antibody on day 7.5 pc wild-type (B) and *Sphk1*^−/−^*Sphk2*^+/−^ (D) uteri treated with Cl-amidine. The middle and lower panels show high-power views of the boxed areas from the corresponding upper panels. Arrows in the lower panels (A–D) indicate dNK cells. Em, embryo. Scale bars in panels A–D represent 500 μm (upper panels) and 50 μm (middle and lower panels). Data are representative of three independent experiments with similar results.

We then examined the expression pattern of ezrin. In day 7.5 pc wild-type mice, ezrin was not expressed in decidual cells in the primary decidual zone surrounding the embryo but was weakly expressed in the secondary decidual zone (SDZ) of the antimesometrial decidua (Figure 4A, middle panel). In the mesometrial decidual region, ezrin was expressed in dNK cells, which were intermixed with mesometrial decidual cells (Figure 4A, lower panel). The presence of dNK cells was confirmed by immunostaining with the anti-DBA antibody (Supplemental Figure 1). Peptidylarginine deiminase inhibition using Cl-amidine treatment significantly increased ezrin expression in decidual cells in the SDZ of the antimesometrial decidua (Figure 4B, middle panel). In day 7.5 pc *Sphk1^−/−^Sphk2^+/−^* mice, ezrin was weakly expressed in the SDZ of the antimesometrial decidua, along with infiltrating neutrophils (Figure 4C, middle panel). In the mesometrial decidua of *Sphk1^−/−^Sphk2^+/−^*mice, ezrin expression was seen in dNK cells, similar to wild-type mice (Figure 4C, lower panel). Cl-amidine treatment reduced decidual cell death in *Sphk1^−/−^Sphk2^+/−^* mice, leading to the appearance of ezrin expression at the fetomaternal interface (Figure 4D, middle panel).

**Figure 5:**
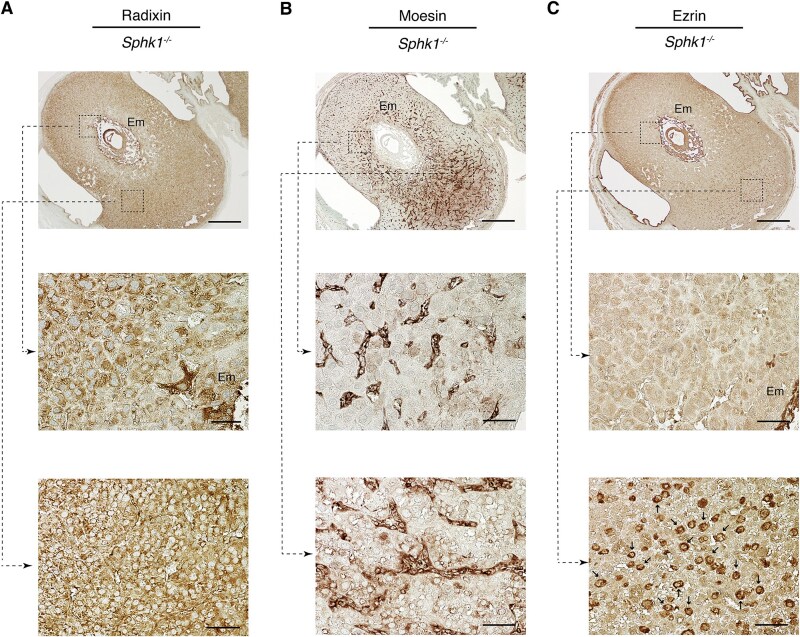
Expression patterns of ERM on day 7.5 pc *Sphk1*^−/−^*Sphk2*^+/+^ uteri. Immunostaining with anti-radixin (A), anti-moesin (B), or anti-ezrin (C) antibodies on day 7.5 pc *Sphk1*^−/−^*Sphk2*^+/+^ uteri. The middle and lower panels show high-power views of the boxed areas from the corresponding upper panels. Arrows in the lower panel (C) indicate dNK cells. Em, embryo. Scale bars in panels A–C represent 500 μm (upper panels) and 50 μm (middle and lower panels). The data represent three independent experiments with similar results.

We also examined the expression patterns of ERM in day 7.5 pc *Sphk1^−/−^Sphk2^+/+^* mice on a mixed genetic background (C57BL/6 × 129Sv), which remain fertile despite moderate defects in decidual cells and blood vessels, as well as elevated sphingoid base levels [12]. Radixin was expressed in decidual cells in both the antimesometrial and mesometrial regions (Figure 5A). Moesin was mainly expressed in endothelial cells, with weaker staining observed in decidual cells (Figure 5B). Ezrin was expressed in decidual cells in the antimesometrial region and in dNK cells in the mesometrial region (Figure 5C). These findings suggest that the expression patterns of ERM in day 7.5 pc decidua from *Sphk1^−/−^Sphk2^+/+^* mice closely resemble those in day 7.5 pc decidua from wild-type mice treated with Cl-amidine.

In summary, we hypothesize that the expression of ERM in the antimesometrial decidual region may be influenced by two factors: citrullination by PADI enzymes and Sphk activity. Citrullination-induced three-dimensional (3D) structural changes may alter the reactivity of these proteins with antibodies, resulting in significant changes in their expression patterns.

### Ezrin, radixin, and moesin are citrullinated exclusively in the decidua during pregnancy

**Figure 6:**
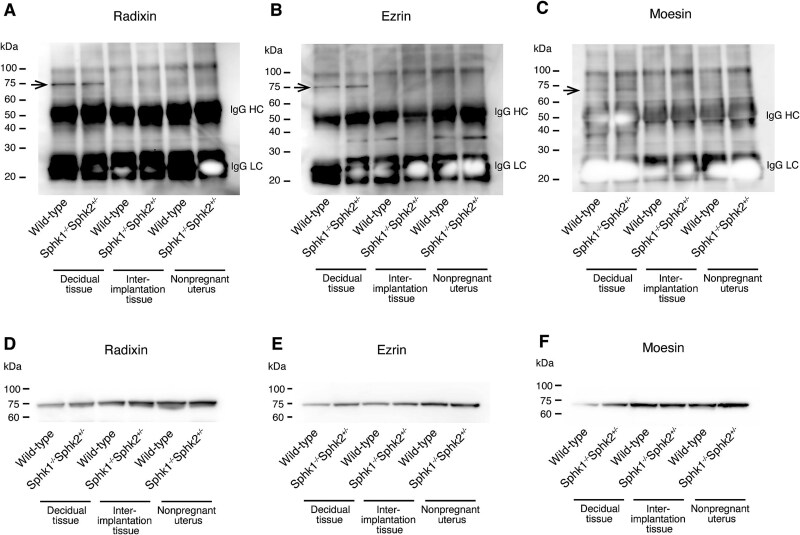
Citrullination of ERM proteins exclusively in the decidua during pregnancy. (A–C) Detection of citrullinated proteins. Tissue homogenates from day 7.5 pc deciduas, day 7.5 pc interimplantation tissues, and nonpregnant uterine tissues of wild-type and *Sphk1^−/−^Sphk2^+/−^* female mice on a mixed genetic background (C57BL/6 × 129Sv) were immunoprecipitated with anti-citrulline antibody and immunoblotted with anti-radixin (A), anti-ezrin (B), or anti-moesin (C) antibodies. Arrows indicate citrullinated proteins. IgG HC, IgG heavy chain; IgG LC, IgG light chain. (D–F) Expression analysis of radixin (D), ezrin (E), or moesin (F) by immunoblotting of the above tissue homogenates.

We further investigated whether radixin, moesin, and ezrin undergo citrullination during pregnancy. Immunoprecipitation followed by immunoblotting was performed on tissue homogenates from day 7.5 pc deciduas, day 7.5 pc interimplantation tissues (uterine tissues without implantation sites), and nonpregnant uterine tissues from wild-type and *Sphk1^−/−^Sphk2^+/−^* female mice on a mixed genetic background (C57BL/6 × 129Sv). Tissue homogenates were immunoprecipitated with an anti-citrulline antibody and then analyzed using anti-radixin, anti-moesin, or anti-ezrin antibodies. Notably, radixin, moesin, and ezrin were citrullinated exclusively in day 7.5 pc deciduas of both wild-type and *Sphk1^−/−^Sphk2^+/−^* female mice, but not in day 7.5 pc interimplantation tissues or nonpregnant uterine tissues from either group of mice (Figure 6A, B, and C). Immunoblotting confirmed that these three proteins were expressed in all uterine tissues examined (Figure 6D, E, and F).

**Figure 7:**
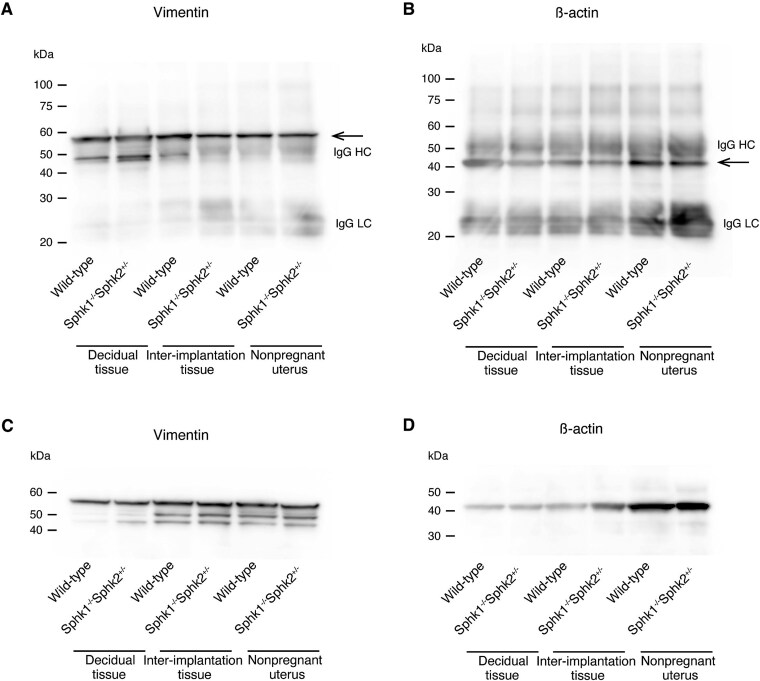
Citrullination of vimentin and ß-actin proteins in uteri. (A and B) Detection of citrullinated proteins. Tissue homogenates from day 7.5 pc deciduas, day 7.5 pc interimplantation tissues, and nonpregnant uterine tissues of wild-type and *Sphk1^−/−^Sphk2^+/−^* female mice were immunoprecipitated with anti-citrulline antibody and immunoblotted using anti-vimentin (A) or anti-ß-actin (B) antibody. Arrows indicate citrullinated proteins. IgG HC, IgG heavy chain; IgG LC, IgG light chain. (C and D) Expression analysis of vimentin (C) or ß-actin (D) by immunoblotting of the above tissue homogenates.

Previous studies have identified several substrates of PADI enzymes, including keratin K1, filaggrin, vimentin, myelin basic protein, glial fibrillary acidic protein, ß-actin, and histone H3. We then compared the citrullination status of vimentin and ß-actin, known substrates of PADI enzymes, with that of ERM in day 7.5 pc deciduas, day 7.5 pc interimplantation tissues, and nonpregnant uteri from wild-type and *Sphk1^−/−^Sphk2^+/−^* female mice. In contrast, vimentin and ß-actin were citrullinated in all uterine tissues examined, regardless of whether they were from pregnant mice or nonpregnant mice (Figure 7A and B). Immunoblotting confirmed the expression of these proteins (Figure 7C and D).

We further investigated whether radixin and ezrin proteins are citrullinated in the uterine tissues of female mice with a C57BL/6 background. Consistent with the findings in female mice on a mixed genetic background (C57BL/6 × 129Sv), radixin and ezrin proteins were citrullinated only in day 7.5 pc deciduas of both wild-type and *Sphk1^−/−^Sphk2^+/−^* female mice with a C57BL/6 background (Supplemental Figure 2A and B). The expression of these proteins was confirmed by immunoblotting (Supplemental Figure 2C and D). This suggests that the citrullination status is not influenced by the mouse genetic background.

Finally, we examined the temporal profile of citrullination status during different developmental stages. Immunoprecipitation followed by immunoblotting was performed on tissue homogenates from day 6.5 pc, day 7.5 pc, day 8.5 pc, and day 9.5 pc deciduas of wild-type and *Sphk1^−/−^Sphk2^+/−^* female mice on a mixed genetic background (C57BL/6 × 129Sv). Radixin was found to be citrullinated in decidual tissues at all developmental stages in both wild-type and *Sphk1^−/−^Sphk2^+/−^* female mice (Supplemental Figure 3). These results suggest a potential role for the citrullination of radixin, ezrin, and moesin in decidualization.

### Ezrin, radixin, and moesin are citrullinated in human decidual tissues

**Figure 8:**
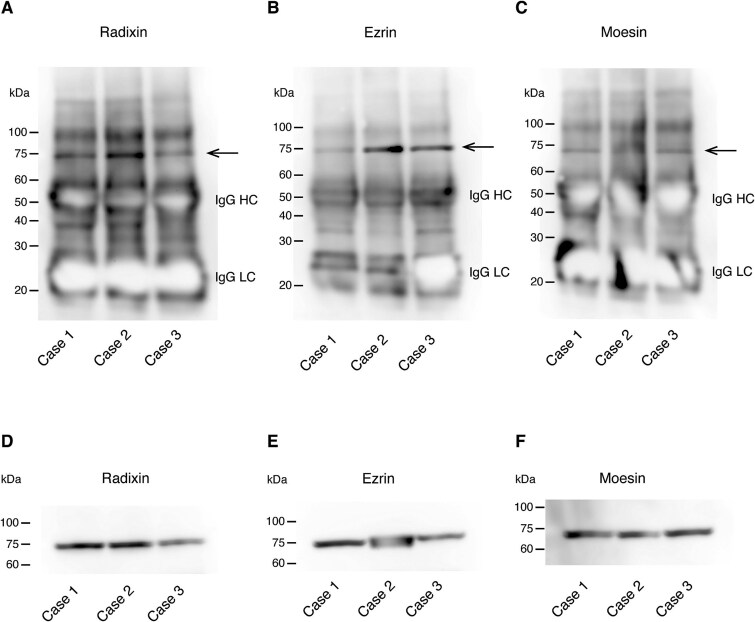
Citrullination of ERM proteins in human decidual tissues. (A–C) Detection of citrullinated proteins. Tissue homogenates from human decidual tissues of three cases with spontaneous abortions or elective terminations of first-trimester pregnancy were immunoprecipitated with anti-citrulline antibody and immunoblotted using anti-radixin (A), anti-ezrin (B), or anti-moesin (C) antibodies. Arrows indicate citrullinated proteins. IgG HC, IgG heavy chain; IgG LC, IgG light chain. (D–F) Expression analysis of radixin (D), ezrin (E), or moesin (F) by immunoblotting of the above tissue homogenates.

We next examined whether ERM proteins are citrullinated in human decidual tissues. Tissue homogenates from human decidual tissues were immunoprecipitated with anti-citrulline antibody and then analyzed by immunoblotting using anti-radixin, anti-moesin, or anti-ezrin antibodies. We studied three cases of spontaneous abortions or elective terminations of first-trimester pregnancy. In all three cases, ERM proteins were significantly citrullinated in the decidual tissues (Figure 8A, B, and C). The expression of these proteins was confirmed by immunoblotting (Figure 8D, E, and F). These findings indicate that citrullination of ERM is a common characteristic of decidual cells in both mice and humans during pregnancy.

### Phosphorylation of ezrin, radixin, and moesin has minimal impact on decidualization

Sphingolipids, S1P and ceramide, are known to influence ERM regulation, with S1P activating ERM by phosphorylating the C-terminal threonine residue [27, 29]. To examine the expression of phosphorylated ERM proteins during pregnancy, we performed immunohistochemical analysis on day 7.5 pc deciduas from wild-type, *Sphk1^−/−^Sphk2^+/+^*, and *Sphk1^−/−^Sphk2^+/−^* mice on a mixed genetic background (C57BL/6 × 129Sv) using anti-ezrin (pThr567)/radixin (pThr564)/moesin (pThr558) antibodies. In all mice analyzed, phosphorylated ERM was expressed in the endothelial cells at the periphery of the decidual tissues (Supplemental Figure 4A, B, and C, middle panels). In certain regions of the antimesometrial decidua, phosphorylated ERM was weakly detected in the plasma membrane of the decidual cells (Supplemental Figure 4A, B, and C, lower panels). We then investigated the expression of phosphorylated ERM proteins by immunoblotting using tissue homogenates from day 7.5 pc deciduas, day 7.5 pc interimplantation tissues, and nonpregnant uterine tissues from wild-type and *Sphk1^−/−^Sphk2^+/−^* female mice on a mixed genetic background (C57BL/6 × 129Sv). In all tissues analyzed, phosphorylated ERM was expressed similarly, regardless of whether the samples were from pregnant or nonpregnant mice (Supplemental Figure 4D). Lastly, we examined the phosphorylation status of ERM proteins in human decidual tissues. Tissue homogenates from three cases of spontaneous abortion or elective termination of first-trimester pregnancies were analyzed by immunoblotting using anti-ezrin (pThr567)/radixin (pThr564)/moesin (pThr558) antibodies. In all three cases, ERM proteins were significantly phosphorylated in the decidual tissues (Supplemental Figure 4E). To further explore the effect of ERM phosphorylation on decidualization, pregnant wild-type mice (C57BL/6 × 129Sv) were treated with anti-ezrin (pThr567)/radixin (pThr564)/moesin (pThr558) antibody on day 4.5 pc or on both days 4.5 and 5.5 pc. Uteri and embryos were evaluated on days 7.5 and 14.5 pc. Histological analysis showed that blocking phosphorylated ERM proteins did not adversely affect decidualization on day 7.5 pc (Supplemental Figure 5A–C). Most embryos in the uteri of antibody-treated wild-type mice were viable and morphologically normal on day 14.5 pc (Supplemental Figure 5D and E). Furthermore, antibody-treated wild-type mice delivered normal pups. These results suggest that blocking phosphorylated ERM proteins does not negatively impact pregnancy. Overall, these findings suggest that the phosphorylation of ERM proteins may have less impact on decidualization during pregnancy compared to their citrullination.

## Discussion

In this study, we showed that ERM proteins are expressed in both decidual and endothelial cells in the decidua during pregnancy, with their expression patterns significantly influenced by two factors: citrullination by PADI enzymes and Sphk activity. We also found that ERM proteins are citrullinated exclusively in the decidua during pregnancy, but not in the interimplantation tissues or nonpregnant uteri in mice. Similar results were observed in human decidual tissues from cases of spontaneous abortions or elective terminations. Our findings contribute to a deeper understanding of the molecular mechanisms involved in early pregnancy.

The PADI gene family consists of five members: PADI1−PADI4 and PADI6. Each PADI enzyme exhibits a distinct tissue distribution and substrate preference. PADI2 is the most widely expressed isoform and is highly conserved across mammalian species. Citrullination by PADI enzymes, which removes the charge from target arginine residues, can significantly alter the protein’s tertiary structure, affect protein–protein and protein–DNA/RNA interactions, and lead to protein unfolding and proteolytic degradation. Peptidylarginine deiminase proteins are notably highly expressed in female reproductive tissues, such as the ovary, uterus, and mammary gland, highlighting their crucial role in female reproduction [30, 31]. In the ovary, PADI6 is predominantly expressed in maturing oocytes and early embryos [32, 33]. Female *padi6* knockout mice are infertile due to cytoskeletal sheet dispersal in oocytes [34], and human *padi6* mutations cause premature embryonic arrest and female infertility associated with absent PADI6 expression in oocytes [35]. These findings highlight the essential role of PADI6 in embryonic development and female fertility. Interestingly, comprehensive transcriptome analysis of mouse tissues showed that the expression levels of PADI1, PADI2, and PADI4 are highest in the uterus among more than 50 mouse tissues examined [31, 36]. Single-cell analyses further show that PADI1, PADI2, and PADI4 are expressed in mouse uterine epithelia, with PADI2 and PADI4 being the predominant isoforms in humans [31, 37, 38]. Furthermore, the role of PADI enzymes in pregnancy has been highlighted in a previous study, where enzymatic activity of PADIs in mouse uterine lysates significantly increased during mid-pregnancy (days 8–10) and gradually decreased in late pregnancy. PADI expression was exclusively found in decidual cells, suggesting a potential role of these enzymes in pregnancy, particularly in decidualization [39]. Together, these studies indicate that PADI enzymes and citrullinated proteins play important roles in female reproductive physiology and may be linked to pregnancy-related disorders. However, despite the identification of citrullinated proteins in various tissues, their functional significance in pregnancy—especially during decidualization—remains poorly understood, aside from a few proteins like citrullinated histones involved in NET formation. Our findings offer important insights into the role of citrullinated proteins during pregnancy.

Both *padi2* and *padi4* single knockout female mice were fertile. Additionally, *padi2*/*padi4* double-knockout female mice did not show significant reproductive defects, while *padi2*/*padi4* double-knockout male mice exhibited several reproductive issues, including delayed puberty, small offspring, reduced testis size, increased apoptosis during spermatogenesis, and lower serum testosterone levels [40]. The lack of significant reproductive defects in PADI2/PADI4-deficient female mice may be due to compensatory mechanisms. Decidualization is a crucial step for a successful pregnancy, as it enables the decidua to form a vascular network for nutrient and gas exchange for the developing embryo before the placenta is fully functional. It also restricts trophoblast invasion and provides immunological protection for the embryo by suppressing the maternal immune responses. Therefore, multiple mechanisms are necessary for proper decidualization and a successful pregnancy. One such mechanism is sphingolipid metabolism. As we previously reported, the sphingolipid metabolic pathway is highly activated in the decidua of mice during normal pregnancy, regardless of genetic background, and disruption of this pathway leads to severe decidual defects and early pregnancy loss. Thus, various strategies, including posttranslational modifications of cytoskeletal components and sphingolipid metabolism, contribute to successful decidualization and pregnancy.

Previous studies have shown that aberrant ezrin functions is linked to female reproductive disorders [41]. In endometriosis, both the expression of ezrin and the Rho pathway were elevated in the ectopic endometrium. Inhibition of ezrin phosphorylation led to reduced expression of the Rho pathway and decreased filopodia formation in ectopic endometrial stromal cells, indicating that ezrin phosphorylation may regulate the migration and invasion of these cells [42]. Furthermore, endometrial tissues from patients with recurrent pregnancy loss (RPL) showed lower levels of both total and phosphorylated ezrin compared to samples from fertile women, as determined by immunoblotting and immunostaining analysis. Furthermore, the actin filament was completely absent in the RPL endometrial samples, suggesting that impaired ezrin expression and activation may disrupt actin binding to the plasma membrane, leading to aberrant cytoskeletal organization in the female reproductive system [43].

To our knowledge, no studies have demonstrated the biological significance of the citrullination of ERM proteins in the physiological and pathological conditions of the female reproductive system. Therefore, a thorough analysis of the role of cytoskeletal component citrullination in decidualization in both mice and humans is an important area for future research.

## Supplementary Material

Suppl_Figure_1_(BOR)_ioaf241

Suppl_Figure_2_(BOR)_ioaf241

Suppl_Figure_3_(BOR)_ioaf241

Suppl_Figure_4_(BOR)_ioaf241

Suppl_Figure_5_(BOR)_ioaf241

## Data Availability

All data generated or analyzed during this study are included in this published article and its supplementary information files.
