# Supplementary material for: Ezrin, radixin, and moesin are novel citrullinated proteins in the decidua during pregnancy
Source: Biol Reprod. 2025 Oct 27;114(3):1018–29. doi: 10.1093/biolre/ioaf241 (PMC13016767; doi:10.1093/biolre/ioaf241)
Supplement: Suppl_Figure_1_(BOR)_ioaf241 [file suppl_figure_1_(bor)_ioaf241.pdf]

Supplemental Figure 1

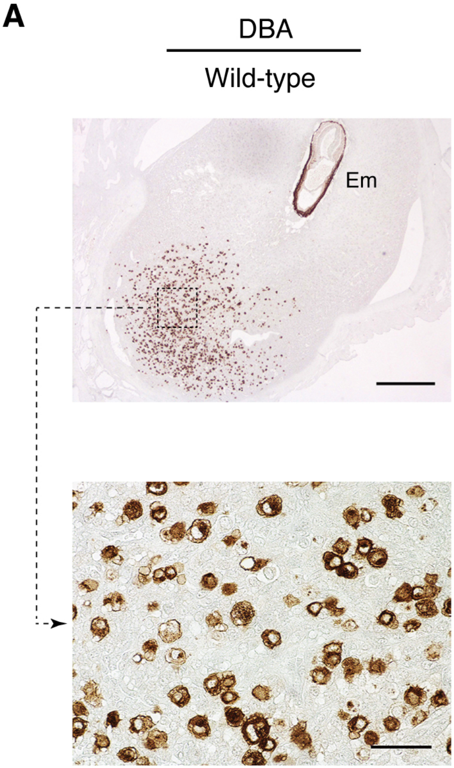

Supplemental Figure 1. DBA expression in the decidua during pregnancy. Immunostaining with anti-DBA antibody on day 7.5 pc wild-type (**A**) uteri. The lower panel shows a high-power view of the boxed area from the upper panel. Em, embryo. Scale bars in **A** represent 500 μm (upper panel) and 50 μm (lower panel). The data represent three independent experiments with similar results.
