# Supplementary material for: Ezrin, radixin, and moesin are novel citrullinated proteins in the decidua during pregnancy
Source: Biol Reprod. 2025 Oct 27;114(3):1018–29. doi: 10.1093/biolre/ioaf241 (PMC13016767; doi:10.1093/biolre/ioaf241)
Supplement: Suppl_Figure_2_(BOR)_ioaf241 [file suppl_figure_2_(bor)_ioaf241.pdf]

Supplemental Figure 2

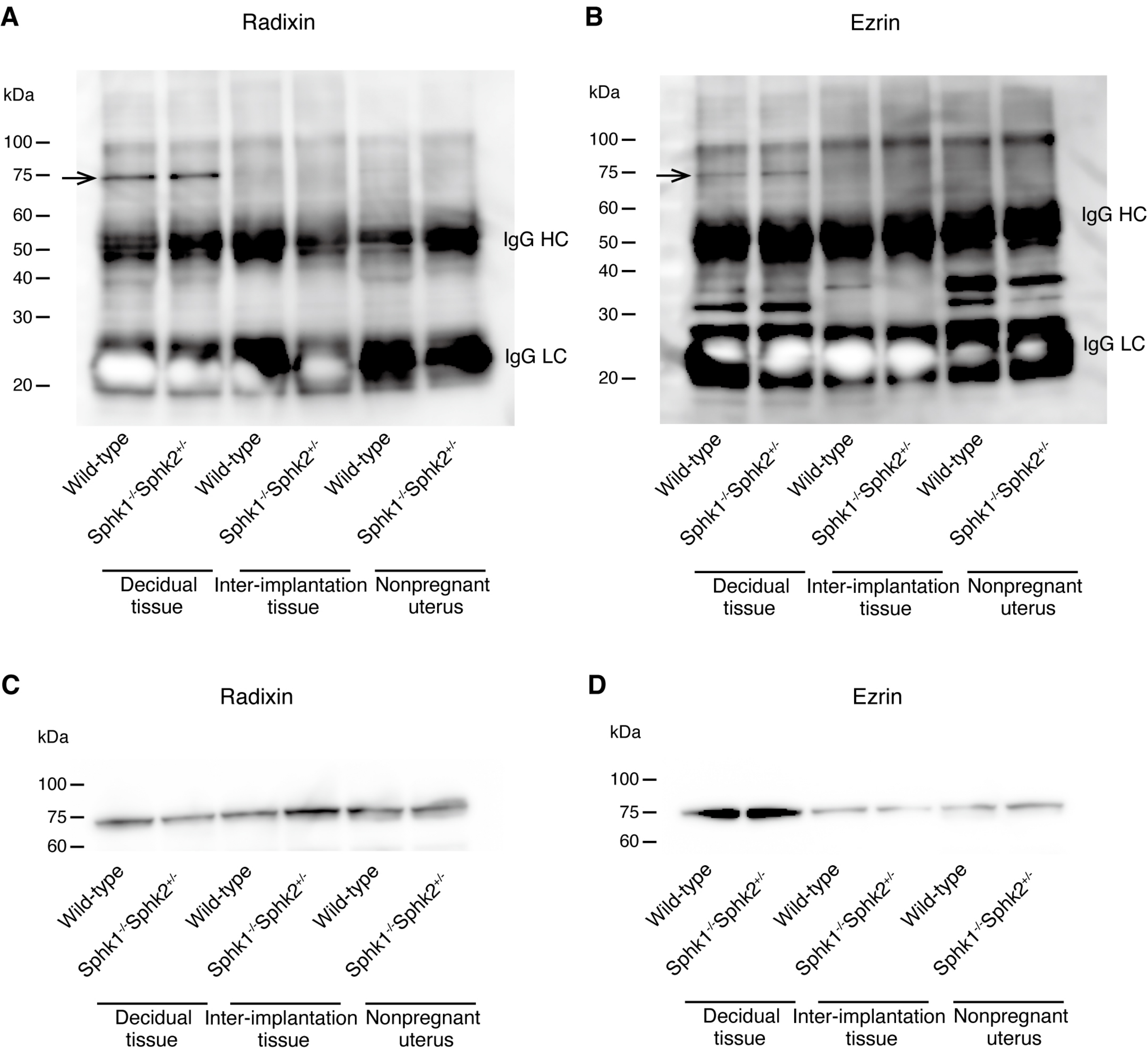

Supplemental Figure 2. Citrullination of radixin and ezrin proteins exclusively in the decidua during pregnancy. (**A** and **B**) Detection of citrullinated proteins. Tissue homogenates from day 7.5 pc deciduas, day 7.5 pc interimplantation tissues, and nonpregnant uterine tissues of wild-type and *Sphk1*$^{-/-}$*Sphk2*$^{+/-}$ female mice on a pure genetic background (C57BL/6) were immunoprecipitated with anti-citrulline antibody and immunoblotted using anti-radixin (**A**) or anti-ezrin (**B**) antibody. Arrows indicate citrullinated proteins. IgG HC, IgG heavy chain; IgG LC, IgG light chain. (**C** and **D**) Expression analysis of radixin (**C**) or ezrin (**D**) by immunoblotting of the above tissue homogenates.
