# Supplementary material for: Ezrin, radixin, and moesin are novel citrullinated proteins in the decidua during pregnancy
Source: Biol Reprod. 2025 Oct 27;114(3):1018–29. doi: 10.1093/biolre/ioaf241 (PMC13016767; doi:10.1093/biolre/ioaf241)
Supplement: Suppl_Figure_3_(BOR)_ioaf241 [file suppl_figure_3_(bor)_ioaf241.pdf]

Supplemental Figure 3

A

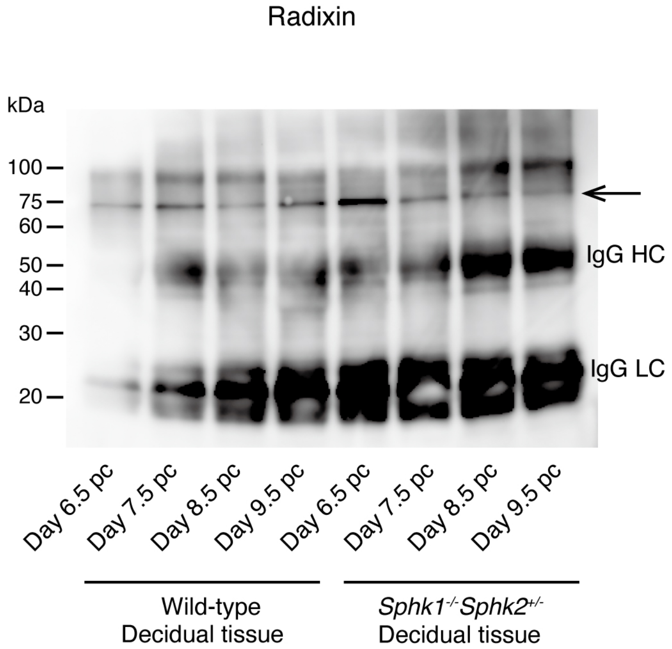

B

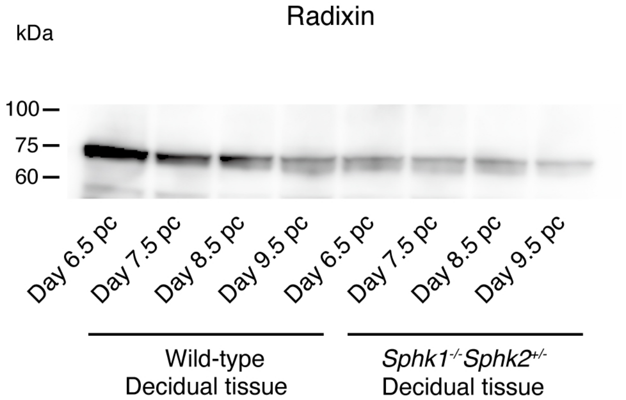

Supplemental Figure 3. Citrullination of radixin in the decidua during various developmental stages of pregnancy. (**A**) Detection of citrullinated proteins. Tissue homogenates from day 6.5 pc, day 7.5 pc, day 8.5 pc, and day 9.5 pc deciduas of wild-type and *Sphk1*$^{-/-}$*Sphk2*$^{+/-}$ female mice on a mixed genetic background (C57BL/6 × 129Sv) were immunoprecipitated with anti-citrulline antibody and immunoblotted using anti-radixin antibody. An arrow indicates the citrullinated protein. IgG HC, IgG heavy chain; IgG LC, IgG light chain. (**B**) Expression analysis of radixin by immunoblotting of the above tissue homogenates.
