# Supplementary material for: Ezrin, radixin, and moesin are novel citrullinated proteins in the decidua during pregnancy
Source: Biol Reprod. 2025 Oct 27;114(3):1018–29. doi: 10.1093/biolre/ioaf241 (PMC13016767; doi:10.1093/biolre/ioaf241)
Supplement: Suppl_Figure_4_(BOR)_ioaf241 [file suppl_figure_4_(bor)_ioaf241.pdf]

Supplemental Figure 4

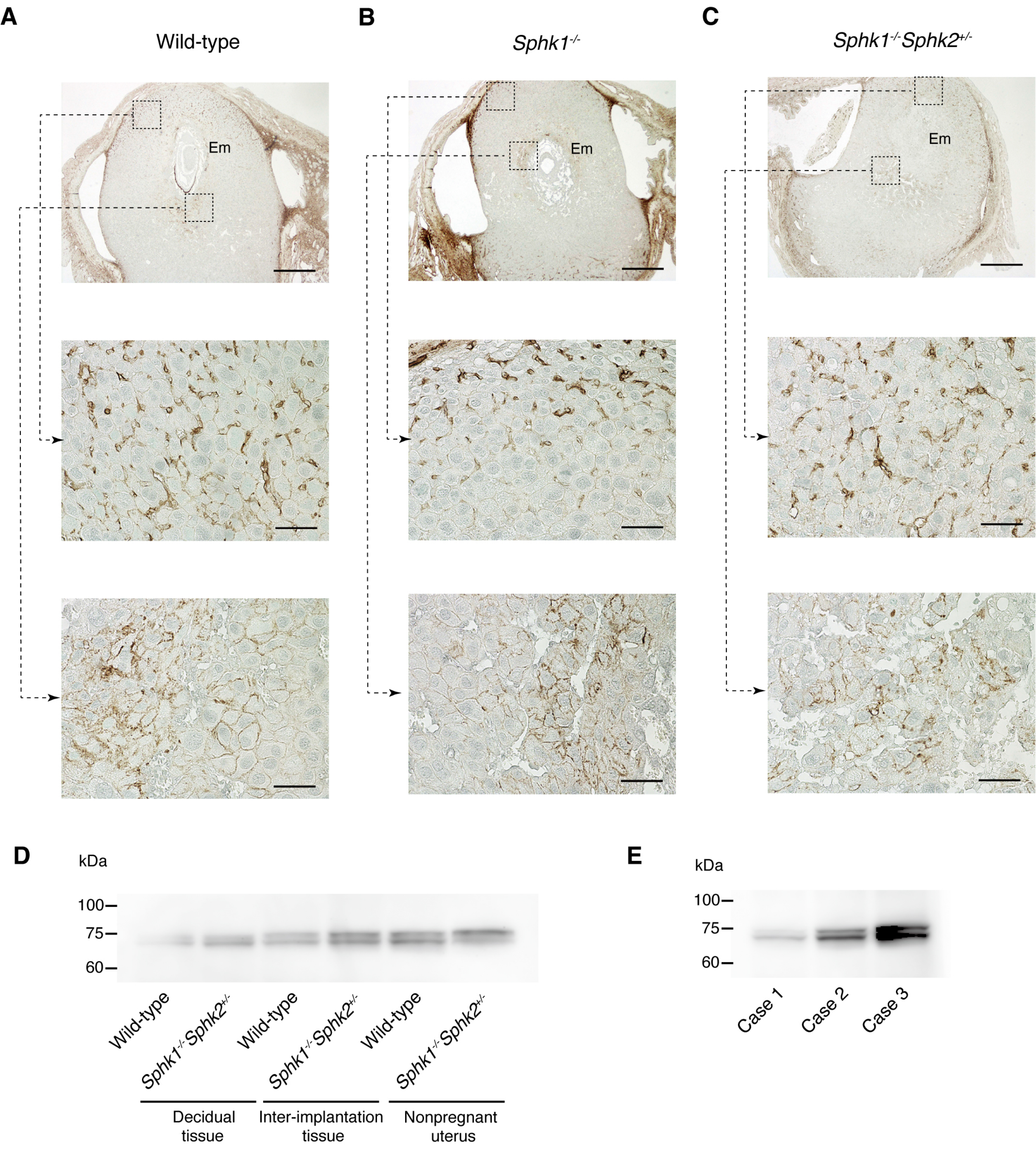

Supplemental Figure 4. Expression of phosphorylated ERM proteins in uteri. (**A**–**C**) Immunostaining with anti-ezrin (pThr567)/radixin (pThr564)/moesin (pThr558) antibody on day 7.5 pc wild-type (**A**), *Sphk1*$^{-/-}$*Sphk2*$^{+/+}$ (**B**), and *Sphk1*$^{-/-}$*Sphk2*$^{+/-}$ (**C**) uteri. The middle and lower panels show high-power views of the boxed areas from the corresponding upper panels. Em, embryo. Scale bars in panels **A**–**C** represent 500 µm (upper panels) and 50 µm (middle and lower panels). (**D**) Expression analysis of phosphorylated ERM by immunoblotting of tissue homogenates from day 7.5 pc deciduas, day 7.5 pc interimplantation tissues, and nonpregnant uterine tissues of wild-type and *Sphk1*$^{-/-}$*Sphk2*$^{+/-}$ female mice. (**E**) Expression analysis of phosphorylated ERM by immunoblotting of human decidual tissues from three cases with spontaneous abortions or elective terminations of first-trimester pregnancies. Data are representative of three independent experiments with similar results.
