# Supplementary material for: Ezrin, radixin, and moesin are novel citrullinated proteins in the decidua during pregnancy
Source: Biol Reprod. 2025 Oct 27;114(3):1018–29. doi: 10.1093/biolre/ioaf241 (PMC13016767; doi:10.1093/biolre/ioaf241)
Supplement: Suppl_Figure_5_(BOR)_ioaf241 [file suppl_figure_5_(bor)_ioaf241.pdf]

Supplemental Figure 5

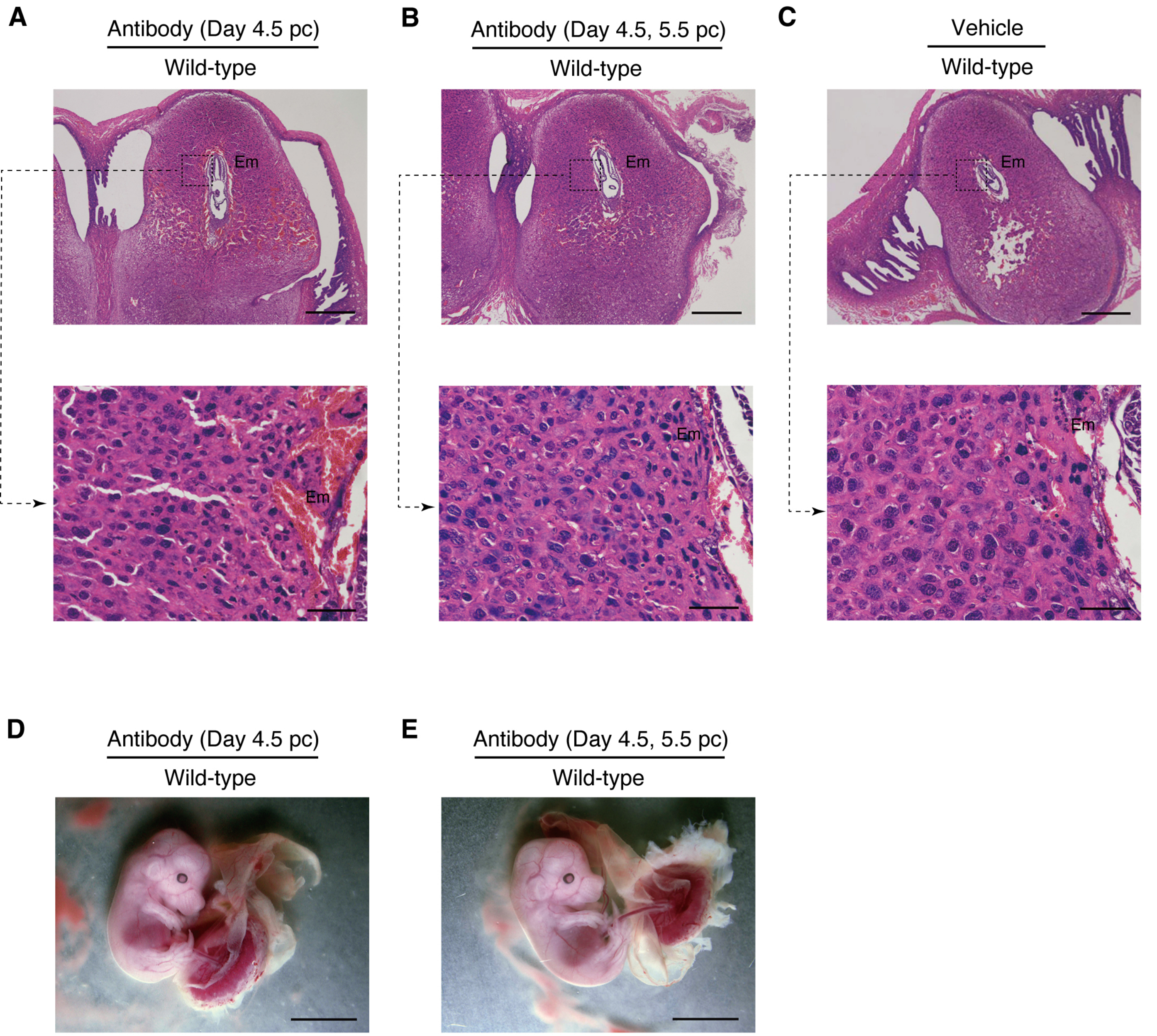

Supplemental Figure 5. Effect of blocking phosphorylated ERM proteins on decidualization. (**A**–**C**) H&E staining of longitudinal sections from day 7.5 pc wild-type uteri. (**A** and **B**) Wild-type female mice were treated with anti-ezrin (pThr567)/radixin (pThr564)/moesin (pThr558) antibody on day 4.5 pc (**A**) or on both days 4.5 and 5.5 pc (**B**). (**C**) Wild-type female mice were treated with vehicle control. The lower panels show high-power views of the boxed areas from the corresponding upper panels. Em, embryo. Scale bars in panels **A**–**C** represent 500 μm (upper panels) and 50 μm (lower panels). (**D** and **E**) Images of uteri and embryos from day 14.5 pc wild-type female mice treated with anti-ezrin (pThr567)/radixin (pThr564)/moesin (pThr558) antibody on day 4.5 pc (**D**) or on both days 4.5 and 5.5 pc (**E**). Scale bars in panels **D** and **E** represent 5 mm.
